# Supplementary material for: Comparison of Methods for Incorporating Related Data When Developing Clinical Prediction Models: A Simulation Study
Source: Biom J. 2026 Aug 27;68(5):e70166. doi: 10.1002/bimj.70166 (PMC13520826; doi:10.1002/bimj.70166)
Supplement: Supplementary file 2 [file BIMJ-68-e70166-s001.zip › third submission code review - R2/reproducibility_supplement/results/Plots/specification_combnationshift_plot_combination_SC_ID8_TS0.25_DS4_model15.pdf]

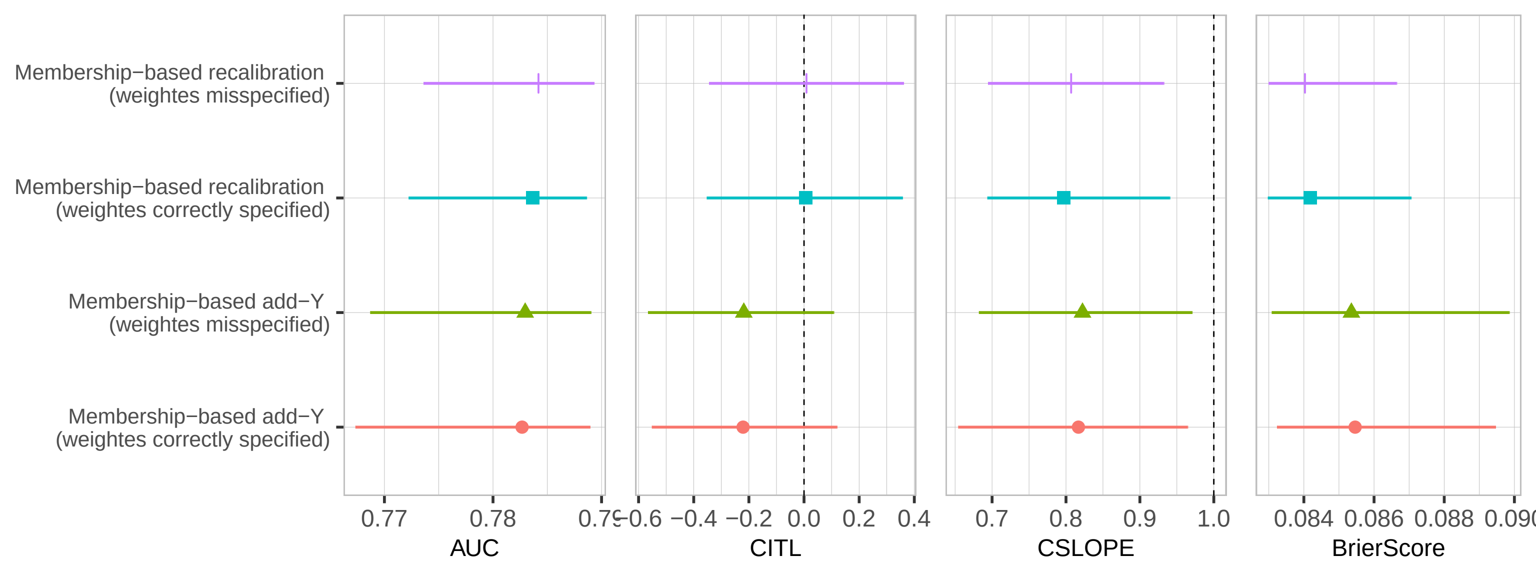

Membership−based recalibration (weightes misspecified)
Membership−based recalibration (weightes correctly specified)
Membership−based add−Y (weightes misspecified)
Membership−based add−Y (weightes correctly specified)
0.77
0.78
0.79
AUC
−0.6
−0.4
−0.2
0.0
0.2
0.4
CITL
0.7
0.8
0.9
1.0
CSLOPE
0.084
0.086
0.088
0.090
BrierScore
